# Characteristics of Adult T-Cell Leukemia/Lymphoma Patients with Long Survival: Prognostic Significance of Skin Lesions and Possible Beneficial Role of Valproic Acid

**DOI:** 10.1155/2015/476805

**Published:** 2015-06-14

**Authors:** Plumelle Yves, Michel Stephane, Banydeen Rishika, Delaunay Christine, Panelatti Gérard

**Affiliations:** ^1^Department of Biological Haematology/Martinique Registry of Haematological Malignancies, Pierre-Zobda Quitman Hospital, University Hospital of Martinique, 97200 Fort-de-France, Martinique; ^2^Martinique Cancer Screening Center, 127 Route de Redoute, 97200 Fort-de-France, Martinique; ^3^Clinical Research and Innovation Unit, Pierre-Zobda Quitman Hospital, University Hospital of Martinique, 97200 Fort-de-France, Martinique; ^4^Department of Internal Medicine, Mangot-Vulcin Hospital, University Hospital of Martinique, 97232 Lamentin, Martinique; ^5^Department of Internal Medicine, Pierre Zobda-Quitman Hospital, University Hospital of Martinique, 97200 Fort-de-France, Martinique

## Abstract

We describe the clinical and biological features of ten patients with a survival superior to ten years (long survival), out of 175 patients diagnosed with Adult T-cell Leukemia/Lymphoma (ATL) in Martinique (1983–2013). There were 5 lymphoma and 5 chronic subtypes. Five of them (3 chronic, 2 lymphoma) had been treated with valproic acid (VA) for neurological disorders developed before or after ATL diagnosis, suggesting a beneficial role for VA as a histone deacetylase inhibitor (HDI) in ATL treatment. Total duration of uninterrupted VA treatment ranged from 8 to 37 years. Overall, the 175 incident ATL cases presented with a median survival of 5.43 months. The five VA-treated (VA^+^) patients presented with longer survival compared to VA treatment-free patients (VA^−^). For chronic subtypes, survival periods were of 213 months for 3 VA^+^ patients and of 33 months for 11 VA^−^ patients (*p* = 0.023). For lymphoma subtypes, survival periods were of 144 months for 2 VA^+^ patients versus 6 months for 49 VA^−^ patients (*p* = 0.0046). ATL cases with skin lesions, particularly lymphoma subtypes, had a longer survival (13.96 months) compared to those without skin lesions (6.06 months, *p* = 0.002). Eight out of the 10 patients presenting with long survival had skin lesions.

## 1. Introduction

The human T-lymphotropic virus 1 (HTLV-1) is the causative agent of ATL (Adult T-cell Leukemia/Lymphoma) [1, 2] and of HAM/TSP (HTLV-1-associated myelopathy/tropical spastic paraparesis), a neurodegenerative disorder [3–5]. The main clinical features of ATL are lymphadenopathy, hepatosplenomegaly, skin lesions, and hypercalcemia. Based on the importance of lymphocytosis, tumor syndrome, hypercalcemia, and lactate dehydrogenase (LDH) values, the Shimoyama classification (Lymphoma Study Group (LSG) classification) recognizes four ATL subtypes: acute and lymphoma aggressive forms, chronic and smoldering indolent forms [6].

The ATL cell, mainly of the T-CD3^low^ CD4^+^ CD7^−^ phenotype, expresses the IL2 alpha receptor (IL2-R, CD25) and presents activation of NF-kappa B in a constitutive manner. Structural and numeric karyotypic abnormalities are frequent [7]. The ATL cell further has an intrinsic resistance to various chemotherapies. All these factors contribute to a very poor prognosis for the ATL disease, with a median survival of 6.2, 10.2, and 24.3 months for acute, lymphoma, and chronic subtypes, respectively [6]. In our series, we however observed patients with a survival superior to ten years. Some of them had received long-term valproic acid (VA) treatment for neurological disorders having developed before or after ATL diagnosis, thus suggesting a beneficial role for VA, as a histone deacetylase inhibitor (HDI), in ATL clinical management.

Two contradictory clinical trials focusing on VA treatment in HAM/TSP patients [8, 9] have ultimately allowed concluding towards the safety of VA use [10] but have also illustrated a lack of clinical improvement in patients [9]. Moreover, the increase, albeit transitory, of proviral load (by clonal expansion) and of viral expression (TAX and GAG-p19) has been described as countering the therapeutic use of VA, because of the risk of aggravation of neuropathy, in TSP/HAM patients. However the blocking of HBZ expression (major actor of ATL leukemogenesis), by VA [10], has hinted at its appropriate use in ATL treatment. Hence, several trials on animal models infected by HTLV-1 or HTLV-1-like retroviruses such as STLV-1 (Simian T-lymphotropic virus type-1) and BLV (bovine leukemia virus) have showed the effectiveness of VA use, alone or associated with antiretroviral drugs [11–14]. In particular, in the absence of cytotoxic treatments, VA induced leukemia/lymphoma regression in a sheep model infected by BLV [11]. Moreover, prolonged survival was achieved in ATL mouse models via the administration of various HDIs (VA, AR-42) [13, 14].

In the field of human lymphoid malignancies, several* in vivo* and* in vitro* studies have described the efficiency of VA and of other HDIs. As a result, different types of HDIs were successfully incorporated into several treatment protocols for other cutaneous T-cell lymphoma unrelated to HTLV-1 [15–18]. One of these HDIs, the PCI-34051, has shown remarkable action specificity on T-cell lines derived from T-cell lymphoma [18]. Apoptosis of various HTLV-1 cell lines, derived from ATL cells, primary ATL cells, or large B-cell lymphoma, was induced* in vitro* by various broad spectrum and specific HDIs [13, 19–21]. Furthermore, VA, AR-42, and LBH589, respectively, reduced the expression of NF-kappa B in HTLV-1 cells [22], serum IL2-R in an ATL mouse model [13, 19], and CCR4 in ATL cells [19]. All of the latter proteins are involved in ATL cell proliferation and invasion.

The objective of this study was to compare the group of ATL patients presenting a survival superior to ten years (long survival) with all ATL patients identified over 30 years in Martinique (1983–2013). We also aim to discuss the rationale of VA use in the treatment of ATL on the basis of current knowledge about the natural history of the integrated HTLV-1 provirus and its tumoral resultant, ATL.

## 2. Methods

### 2.1. Patient Population

This retrospective analysis concerns all ATL patients identified by the Martinique Registry of hematological malignancies and diagnosed between 1st January 1983 and 31st March 2013. Identification of patients, treated with VA, was the combined resultant of the retrospective analysis of patients diagnosed before year 2000 and of the prospective monitoring of patients as from year 2000. All study subjects presented a typical clinical presentation of the ATL disease (with histological and/or immunological confirmation) and had a positive serology for the HTLV-1 virus. Clinical subtypes were classified according to the LSG criteria [3]. Patients unanimously received treatment at the University Hospital of Fort-de-France. Five patients further received VA at daily doses of 500 mg orally.

### 2.2. Statistical Analysis

Continuous variables (age at diagnosis) are shown as mean ± SD and were compared by Student's unpaired *t*-test. Categorical variables, such as gender, tumor syndrome (lymphadenopathy, hepatomegaly, splenomegaly, and skin lesion), calcium and LDH values, eosinophilia status, and* Strongyloides stercoralis* (Ss) infection status, are presented as counts and percentages and were compared with the Fisher exact test. Survival curves were generated by the Kaplan-Meier method, and intergroup survival (according to ATL type, presence/absence of skin lesions, and VA treatment) was compared with the log-rank test. For all survival analyses, patients were considered at risk until date of death or date of last contact. Patients lost to follow-up were considered at risk until the date of last contact, at which point they were censored. Probability was significant at a level of 0.05 and all statistical tests were 2-tailed. Statistical analysis was performed with the use of the SAS 9.2 software (SAS Institute Inc., Cary, NC, USA).

## 3. Results

During the study period, 175 new cases of ATL were identified. Clinical data are summarized in Table 1. The median age at diagnosis was 56 years (16–95 years), with a sex ratio of 1.01. The distribution of acute, chronic, and lymphoma subtypes was 62.9%, 29.1%, and 8%, respectively. No smoldering type was identified.

**Figure 1:**
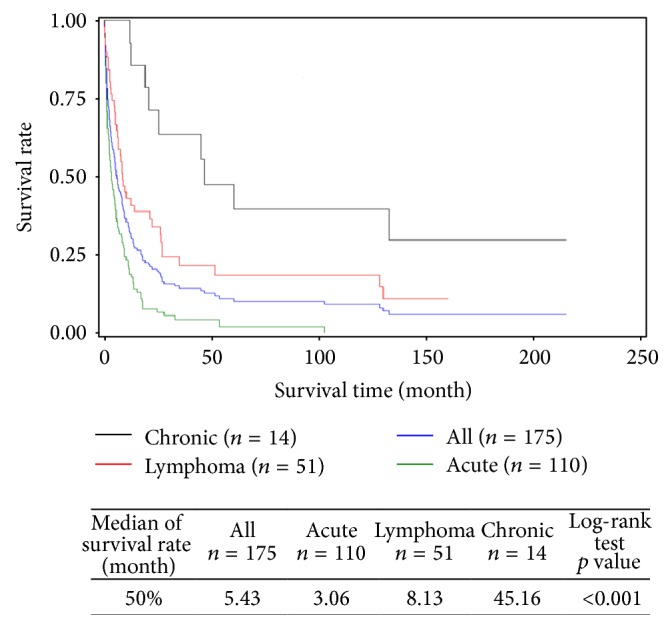
Global and specific survival, by clinical subtypes (acute, lymphoma, and chronic), of Adult T-cell Leukemia/Lymphoma (ATL) patients diagnosed between 1st January 1983 and 31st March 2013 in Martinique (*N* = 175).

**Figure 2:**
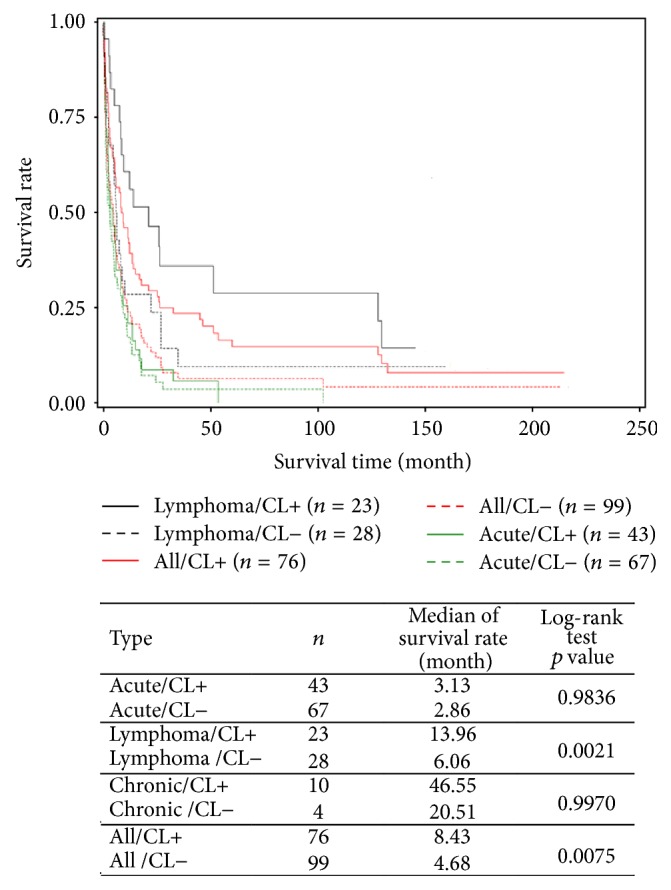
Global and subtype-specific survival (acute, lymphoma, and chronic), according to presence of cutaneous lesions (CL+) or absence of cutaneous lesion (CL−), of Adult T-cell Leukemia/Lymphoma (ATL) patients diagnosed between 1st January 1983 and 31st March 2013 in Martinique. Because of the small number of patients, the survival curves of chronic* subtypes* are not drawn.

In all, 154 deaths were registered during the study period and 7 patients were lost to follow-up. Median survival was 5.43 months for all cases: 3.06 months for those presenting with acute subtypes, 8.13 months for lymphoma subtypes, and 45.16 months for chronic subtypes (Figure 1). For lymphoma types, patients with skin lesions registered a significantly longer survival compared to patients without skin lesions (13.96 months versus 6.06 months, *p* = 0.002) (Figure 2).

In our study, 10 patients (4 men and 6 women) presented with a long survival (superior to 10 years) (Table 2). The median age for long survival patients was of 45 years, with the following clinical ATL subtype distribution: 5 lymphoma and 5 chronic types including one patient with an exclusively cutaneous form (patient 7). Eight patients presented with skin lesions and one patient was diagnosed with lung nodules (patient 2) while another one suffered from acute pancreatitis, ascites, and pleural effusion (patient 4). The clinical characteristics of these patients are compared to the whole ATL patient sample in Table 1. Overall, patients with long survival were younger (*p* = 0.017) and presented with a higher frequency of skin lesions (*p* = 0.016) when compared to the rest of the patient sample.

Two patients were described with hypercalcemia and four patients presented with elevated LDH values while four other patients had eosinophilia (Table 2). A monoclonal integration of the HTLV-1 provirus was detected in patients 7, 9, and 10 (other patients were not tested). A karyotype revealed a hyperploidy with clonal numeric and structural karyotypic abnormalities in 2 patients (patients 2 and 8) and chromosomal deletions in a third one (patient 3).

Patient medical history and treatment course are presented in Table 3. Overall, three deaths related to ATL disease were observed during the study period (patients 6, 9, and 10). ATL treatment for chronic forms consisted in a combination of interferon alpha (IFN) and antiretroviral drugs. Lymphoma subtypes received a CHOP (cyclophosphamide, hydroxydaunorubicin, oncovin, and prednisolone) treatment, sometimes associated with etoposide, and followed or not by an association of IFN and antiretroviral drugs. Two patients received no treatment at all. Five patients (1 to 5) received VA for neurological disorders. Patients 1 and 5 were treated with VA, without interruption, for 20 and 15 years before ATL onset, respectively, for epilepsy and craniostenosis with congenital blindness. Patients 2, 3, and 4 received VA treatment 3 years after ATL diagnosis for depressive syndrome, chronic psychosis (against an epileptical background), and disabling neuropathy, respectively. All five patients (patients 1 to 5) continued with VA treatment without interruption after ATL diagnosis and parallel to ATL treatment. The total duration of VA treatment ranged from 8 to 37 years (Table 4). The five VA-treated (VA^+^) patients presented with longer survival compared to VA treatment-free patients (VA^−^). For chronic subtypes, survival periods were of 213 months for 3 VA^+^ patients versus 33 months for 11 VA^−^ patients (*p* = 0.023). For lymphoma subtypes, survival periods were of 144 months for 2 VA^+^ patients and of 6 months for 49 VA^−^ patients (*p* = 0.0046). For 2 of these 5 patients, other family members also developed an ATL condition with survival spanning from 3 days to 2 years. These family members did not receive any VA treatment. It is to be noted that no other patient from our series, apart from the five identified ones, received VA.

## 4. Discussion

ATL is of very poor prognosis and the current urgency is finding treatments prolonging survival. Recently, an international meta-analysis revealed the efficacy of associating IFN with zidovudine in leukemia and chronic subtypes [23]. This finding was later confirmed by another study for lymphoma subtypes, combining IFN with chemotherapy [24]. However, relapses still remain the rule for aggressive forms of ATL and long survival periods are rare [25, 26]. In our patient series, observed characteristics such as age, sex ratio, tumoral syndrome, hypercalcemia, LDH rates, eosinophilia (Table 1), and median survival according to clinical ATL subtypes (Figure 1) were comparable to what is described by other authors [6]. Only the frequency of skin lesions and early age significantly distinguished long survival patients (>10 years) from the rest.

During the diagnosis period of patients with long survival, high LDH (4 patients) or high serum calcium rates (2 patients) showed that these prognostic tumoral markers were not barriers against an indolent evolution. The same could apply to eosinophilia [27, 28] and abnormal karyotype [7] which are considered as unfavorable prognostic factors. The fact that none of the ten long survival patients showed acute type ATL during diagnosis period suggested that the indolent nature, characterized by lymphoma or chronic presentation, either was intrinsically linked to certain ATL cells (patients 6 to 10) or was acquired through a stabilizing treatment such as VA (as seen in 5 of our patients, patients 1 to 5). Lymphoma types, observed in 5 out of 10 patients with long survival, showed that this clinical form, regarded as aggressive, did not prevent an indolent evolution either. However, a recent report on 90 indolent ATLs (65 chronic and 25 smoldering types) described a faster than expected growth, which was further accelerated by conventional cytotoxic agents, thus highlighting the need for careful clinical monitoring of these subtypes and for the development of targeted treatment courses [26], including new antiretroviral therapy in combination with interferon [23]. Therefore, the rapid evolution of some smoldering forms not only could explain their absence in our patient series but could also be the cause of their delayed diagnosis at more severe stages. Moreover, the hypothesis of an inefficient screening to identify smoldering forms cannot be excluded.

Furthermore, in the patient group with long survival, the high proportion of patients with skin lesions and early age at diagnosis suggests that skin tissue could be a primary, precocious, and privileged location for the ATL cell. Infiltration of other tissues would therefore be secondary, delayed, and metastatic in nature. Several facts underlie this hypothesis as follows: (i) the existence of a rare form of exclusively cutaneous ATL [29] as presented by the youngest patient of our series (patient 7), (ii) the observation of multisystem damage in acute forms at diagnosis, and (iii) the significantly longer survival of patients with skin lesions (particularly of the lymphoma subtype) in our overall patient series. These observations suggest that the early treatment of skin lesions in indolent ATL forms could limit evolution towards aggressive forms. However, optimal treatment for these forms remains to be determined. As reported recently, combination therapy with antiretroviral drugs and arsenic [30], or anti-CCR4 antibodies, alone or in combination with other agents [31], could be attractive alternatives to chemotherapy.

As described by their medical history, one patient of our series received immunosuppressants such as antilymphocyte serum, cyclosporine, and corticosteroids due to kidney transplant during the 5 years preceding ATL disease while another received IFN, with antiviral and antiproliferative effects, for hepatitis C treatment. These treatments, especially antilymphocyte serum, cyclosporine, and IFN, could alter the integrity of the ATL cell, inducing apoptosis or repressing tumor extension through their anti-inflammatory effects and thus contributing to a slow evolutionary process [32]. In two patients (patients 4 and 5), VA could have stimulated apoptosis in ATL cells treated with a CHOP regimen, as what was described on B lymphoma cell lines treated with the same regimen [20].

It is to be noted that, due to the small patient sample treated with VA, their longer survival periods (as compared to untreated patients) must be considered with care. Moreover, the introductory modalities of VA in these 5 patients were not homogeneous: two patients received VA for more than 15 years before ATL diagnosis while the remaining three only received VA, three years after ATL diagnosis, thus pointing towards the fact that VA administration before ATL treatment might not be necessary for its probable beneficial role. All 5 patients (patients 1 to 5) continued VA treatment without interruption and parallel to ATL treatment for time periods varying between 8 and 37 years. This observation might emphasize the importance of continuous VA administration over long time periods. The necessity for uninterrupted VA treatment was further underlined in a test on an ATL/NOD-SCID mouse model treated by another HDI, AR-42 [13], and in a clinical trial focusing on leukemia/lymphoma treatment by VA in sheep infected by BLV [11].

However, these previous studies are not informative about VA's action on the integrated HTLV-1 provirus. The provirus, integrated in the host cell's genome, is flanked by two repeating units, the “long terminal repeat” (LTR), that is, the viral promoters (5′LTR and 3′LTR) which control the expression of viral genes, including* TAX* and HTLV-1 bZIP gene (*HBZ*). The HBZ protein might inhibit transactivation controlled by the TAX protein, which is itself under control of the 5′LTR [33, 34]. The action mechanism of HDs, at the level of both viral LTR promoters, could consist in a mutually exclusive and therefore transient and reversible bond between the TAX protein (or HDs) with viral LTR promoters [35]. VA and other HDIs, such as trichostatin (TSA), by inhibiting HDs, could promote the binding of TAX on LTRs which when activated trigger the transcription of HTLV-1 genes such as* TAX* [36]. This renders HTLV-1 cells susceptible to cytotoxic attack by the immune system. However, this action was found to vary according to HDI type [11, 13, 19], suggesting specific actions by each HDI variant.

The two LTRs are virtually identical in terms of DNA sequence. At their level, they present an almost equal distribution of activators and coactivators, suggesting a common functionality [35]. However, they diverge in terms of viral transcriptional activity which is dependent on histone environment [19, 35, 37–39]. This environment consists of histones and enzymes comprising the whole HD family and histone acetyltransferases. The observation of a preferential localization of HDs 1 and 2 (at the level of the 5′LTR) and HD 3 (at the level of 3′LTR) [35, 36] certifies a specific histone environment for each LTR [35, 37], thus resulting in a differential transcriptional activity. The greater acetylation of H3 and H4 histones of the proviral genome, for which acetylation is induced by HDIs such as TSA and sodium butyrate [37] or LBH589 [19], further strengthens this point of view and prompts to seek HDIs with specific action on each LTR.

Moreover, the entire cellular genome has a histone environment. Therefore, HDIs, such as VA, could restore the expression of many switched-off genes, particularly those that encode CD3/TCR protein complex. It could also be the case for the CD7 protein which is operatively associated with the CD3/TCR complex and which has a defective expression in ATL cells [40–42]. In this manner, by resetting the physiological expression of the CD3/TCR/CD7 complex, HDIs could strengthen the body's defenses against TAX-expressing cells. Restoration by TSA, of the expression of the tumor suppressor gene,* NDRG2* (N-myc downstream-regulated gene 2) on ATL cells [43], is an example of the potential action of HDIs on cellular genes.

Furthermore, the high frequency of aberrantly hypermethylated cellular genes in the ATL cell, involved in cell growth or apoptosis [44], suggests that the association of HDIs with hypomethylating agents, such as 5-aza-2′-deoxy-cytidine, could normalize their transcription. However, due to the risk of the hypertranscription of the* HBZ* with proliferative potential [34, 45, 46], the selective methylation of the 5′LTR [38, 47] or its deletion [48] observed in HTLV-1 cells counters the use of hypomethylating agents in ATL treatment. The existence of a synergistic effect on the demethylation of the 5-aza-2′-deoxy-cytidine/TSA combination also leads to the same conclusion [49].

Moreover, in healthy carriers, HDI-induced TAX overexpression, due to its mutagenic effects, could favor early immortalization of HTLV-1 clones. This has been observed* in vitro* with HTLV-1 cells in culture and in transgenic animal models for* TAX* [50, 51]. On the other hand, in indolent ATL types, TAX overexpression could induce the appearance of more aggressive clones [52]. In contrast, the risk of inflammatory conditions, induced by TAX overexpression, must also be weighed in patients with a functional 5′LTR. Thus, the combination of a reverse transcriptase inhibitor (such as azidothymidine) to a HDI (such as VA) seems necessary to prevent viral spread like that observed in baboons naturally infected with STLV-1, a retrovirus close to HTLV-1 [12]. Interestingly, in the primate model, reduced proviral load, following this combination therapy, was associated with an increase of CD8 cells probably with an antiretroviral activity [12].

It is reasonable to think that, to be active, HDIs require intact provirus and its histone environment. However, ATL patients and healthy carriers frequently have genetic or epigenetic abnormalities at the 5′LTR level [38, 47, 48]. As a result, the therapeutic use of HDIs needs to be preceded by a detection phase of genetic abnormalities in the proviral genome and its histone environment to identify good responders to inhibitors of histone enzymes, probably in combination with antiretroviral drugs.

## 5. Conclusion

The long survival of ATL patients, recipients of long-term uninterrupted VA treatment, is an incentive to consider HDIs as potentially effective agents in ATL treatment, for induction or as a maintenance therapy either alone or in combination with other molecules. As such, HDIs are not curator agents but are stabilizing agents in indolent ATL forms. The asymmetric risk, resulting from the differential effects of HDIs at the level of the 2 LTRs, prompts to seek HDIs with specific action on each LTR. The use of HDIs also requires thought about the correct choice of associated molecules, with the objective of creating synergy without viral spread and for determining the administration sequence of those associated molecules. The selection of high risk candidates, such as ATL patient relatives, healthy carriers, or Ss-infected persons, is also a point to focus on. Furthermore, in the patient group with long survival, the high proportion of patients with skin lesions suggests that skin tissue could be a primary, precocious location for the ATL cell and that their early treatment in indolent ATL forms could limit evolution towards aggressive forms.

## Figures and Tables

**Table 1:** Clinical characteristics, according to survival periods, of Adult T-cell Leukemia/Lymphoma (ATL) patients, diagnosed between 1st January 1983 and 31st March 2013 in Martinique.

	Mean age ± SD^Δ^ (years)	Men (%)	ADP^*∗*^ (%)	HM^†^ (%)	SM^‡^ (%)	CL^§^ (%)	Ca^||^ (%)	LDH^#^ > 2N (%)	Ss^*∗∗*^ (%)	Eos^*∗∗∗*^≥ 1 × 10^9^/L (%)
All patients (*N* = 175)	56.9 ± 17.0	50.3	80.0	21.7	18.3	43.4	46.9	55.4	42.5	16.0

Patients not presenting with long survival (*N* = 165)	57.5 ± 16.8	50.9	81.2	22.4	19.4	41.2	48.5	56.4	43.4	14.9
Patients presenting with long survival (>10 years) (*N* = 10)	44.0 ± 14.5	40.0	60.0	10.0	0.0	80.0	20.0	40.0	30.0	40.0
*P* ^*α*^	**0.017**	0.760	0.104	0.356	0.125	**0.016**	0.077	0.313	0.559	0.038

^*∗*^ADP: adenomegaly, ^†^HM: hepatomegaly, ^‡^SM: splenomegaly, ^§^CL: cutaneous lesions, ^||^Ca: hypercalcemia, ^#^LDH: lactate dehydrogenase, ^*∗∗*^Ss: *Strongyloides stercoralis*, ^*∗∗∗*^Eos: eosinophilia, and ^Δ^SD: standard deviation; ^*α*^ *P*: level of significance set at 5%.

**Table 2:** Clinical and biological characteristics of Adult T-cell Leukemia/Lymphoma (ATL) patients, diagnosed between 1st January 1983 and 31st March 2013, presenting with a survival superior to 10 years (long survival) in Martinique.

Patient	Age (years)	Gender^*∗*^	ATL type^†^	ADP^‡^	SM^§^	HM^||^	CL^#^	Ss^*∗∗*^	Hb^*∗∗∗*^	PN^Δ^	Plt^*α*^	Lym^*β*^	AbN lym^*γ*^ (%)	Ca^*δ*^	LDH^*ε*^	Eos^*ζ*^
1	46	F	chr	+	−	−	−	+	11.7	3,1	192.0	10,1	58.0	−	1.0	−
2	40	F	chr	−	−	−	+	−	13.6	10,9	254.0	174,0	89.0	−	1.0	+
3	46	F	chr	−	−	−	+	−	7.3	4.3	NK	4,1	NK	−	2.1	−
4	50	M	lym	+	−	−	+	+	13.0	6.0	230.0	1,5	0.0	−	1.9	+
5	34	M	lym	+	−	−	−	−	9.7	0.9	127.0	1,5	0.0	+	2.6	−
6	62	F	lym	−	−	+	+	−	7.8	1.0	232.0	3,7	0.0	−	4.6	+
7	16	F	chr	−	−	−	+	−	11.5	2.0	N	3,6	9.0	−	1.0	−
8	36	M	lym	+	−	−	+	−	12.1	4,5	153.0	1,7	0.0	+	4.1	−
9	68	M	chr	+	−	−	+	+	12.9	3.6	154.0	2.4	33.0	−	1.1	+
10	45	F	lym	+	−	−	+	−	N	N	N	N	0.0	−	1.0	−

^*∗*^M: male, F: female, ^†^chr:chronic – lym: lymphoma,^‡^ADP: adenomegaly, ^§^SM: splenomegaly, ^||^HM: hepatomegaly, ^#^CL: cutaneous lesions, ^*∗∗*^Ss: *Strongyloides stercoralis*, and ^*∗∗∗*^Hb: hemoglobin (g/dL).

^Δ^PN: polynuclear cells (1 × 10^9^/L), ^*α*^Plt: platelet (1 × 10^9^/L), ^*β*^Lym: lymphocytes (1 × 10^9^/L), ^*γ*^AbN lymph: abnormal lymphocytes (% of atypical lymphocytes relative to total lymphocytes), ^*δ*^Ca: hypercalcemia, ^*ε*^LDH: lactate dehydrogenase (expressed as multiples of the normal value), ^*ζ*^Eos: eosinophilia (1 × 10^9^/L), +: presence, −: absence, N: within normal range, and NK: not known.

**Table 3:** Medical history, date of diagnosis, and treatment course of neurological disorders of Adult T-cell Leukemia/Lymphoma (ATL) patients, diagnosed between 1st January 1983 and 31st March 2013, presenting with a survival superior to 10 years (long survival) in Martinique.

Patient	Age (years)	Gender^*∗*^	Medical history (time period)	Neurological disorders (time period)	Start of valproic acid (VA) treatment	ATL clinical subtype (diagnosis date)	ATL treatment	Survival period at 31st March 2013 (months)
1	46	F	Neo-uterus (1983)	Epilepsy (1975)	1975	Chronic (1995)	IFN^†^, Hivid	216

2	40	F	Chickenpox	Depressive syndrome (2004)	2004	Chronic (2001)	IFN^†^, combivir-VP16^#^	142

3	46	F		Chronic psychosis with an epileptical background (1996)	1999	Chronic (1996)	—	205

4	50	M	Inguinal hernia, alcoholism	Disabling neuropathy (2004)	2004	Lymphoma (2001)	CHOP^‡^, IFN^†^-VP16^#^, Hivid, combivir	128

5	34	M	Posttransfusion hepatitis C (2000)/IFN^†^	Craniostenosis/surgery (1985)	1985	Lymphoma (2000)	CHOP^‡^, VP16^#^, Hivid	160

6	62	F	Hookworm disease	No	—	Lymphoma (2000)	—	129 (deceased)

7	16	F		No	—	Chronic (2000)	VP16^#^, combivir, IFN^†^, rituximab	150

8	36	M	Perinatal exchange transfusion, malaria, smoking, alcoholism	No	—	Lymphoma (2002)	VP16^#^, COPP^§^, COPADM^*∗∗*^, MTX, AZT, IFN^†^, combivir	132

9	68	M	Erythroderma (1993), strongyloidiasis (1994), alcoholism	No	—	Chronic (2000)	CHOP^‡^, VP16^#^	132 (deceased)

10	45	F	Hypertension, kidney failure (1988), kidney transplant on nephroangiosclerosis (1990), IgG^||^ kappa peak	No	—	Lymphoma (1995)	CHOP^‡^, MTX^*∗∗∗*^, caryolysine, Epivir, photochemotherapy, radiotherapy	130 (deceased)

^*∗*^M: male, F: female, ^†^IFN: interferon, ^||^IgG: immunoglobulin G, ^‡^CHOP: cyclophosphamide, hydroxydaunorubicin, oncovin, and prednisolone, ^§^COPP: cyclophosphamide, oncovin, procarbazine, and prednisolone, ^#^VP16: vepezide 16, ^*∗∗*^COPADM: cyclophosphamide, vincristine, prednisolone, doxorubicin, and methotrexate, ^*∗∗∗*^MTX: methotrexate, ^Δ^AZT: azidothymidine, Hivid/combivir: nucleoside analog reverse transcriptase inhibitor, rituximab: monoclonal antibody anti-CD20, caryolysine: chlormethine chlorhydrate, and Epivir: doxorubicine chlorhydrate.

**Table 4:** Duration of valproic acid (VA) treatment before and after Adult T-cell Leukemia/Lymphoma (ATL) in patients diagnosed between 1st January 1983 and 31st March 2013 presenting with a survival superior to 10 years (long survival) in Martinique.

Patient	VA before ATL onset (years)	VA after ATL onset (years)	Total VA treatment duration (years)
1	20	17	37
2	0	8	8
3	0	13	13
4	0	8	8
5	15	12	27
